# Fabry-Stroke Italian Registry (FSIR): a nationwide, prospective, observational study about incidence and characteristics of Fabry-related stroke in young-adults. Presentation of the study protocol

**DOI:** 10.1007/s10072-021-05615-2

**Published:** 2021-10-05

**Authors:** Ilaria Romani, Patrizia Nencini, Cristina Sarti, Giovanni Pracucci, Marialuisa Zedde, Antonia Nucera, Vittoria Cianci, Jessica Moller, Danilo Toni, Daniele Orsucci, Carmela Casella, Vincenza Pinto, Pasquale Palumbo, Leonardo Barbarini, Rita Bella, Michele Ragno, Umberto Scoditti, Domenico Maria Mezzapesa, Rossana Tassi, Marina Diomedi, Anna Cavallini, Gino Volpi, Alberto Chiti, Guido Bigliardi, Simona Sacco, Giovanni Linoli, Stefano Ricci, Antonello Giordano, Bruno Bonetti, Maurizia Rasura, Emanuela Cecconi, Lucia Princiotta Cariddi, Roberto Currò Dossi, Marta Melis, Domenico Consoli, Donata Guidetti, Silvia Biagini, Donatella Accavone, Domenico Inzitari

**Affiliations:** 1 Department of Neurosciences, Psychology, Pharmacology, and Child Health (NEUROFARBA), University of Florence, Florence, Italy; 2 Stroke Unit, Careggi University Hospital, Firenze, Italy; 3 Neurology, S. Maria Nuova Hospital, Reggio Emilia, Italy; 4 Neurovascular Treatment Unit, Spaziani Hospital, Frosinone, Italy; 5 Neurology, Bianchi-Melacrino-Morelli Hospital, Reggio Calabria, Italy; 6 Neurology-Stroke Unit, Brotzu Hospital, Cagliari, Italy; 7 Emergency Department Stroke Unit, Umberto I Polyclinic Hospital, Sapienza University of Rome, Rome, Italy; 8 Neurology, Lucca Hospital, Lucca, Italy; 9 Stroke Unit, Department of Clinical and Experimental Medicine, AOU Policlinico G. Martino, University of Messina, Messina, Italy; 10 Neurology and Stroke Unit, Di Summa - Perrino Hospital, Brindisi, Italy; 11 Neurology, Neurophysiopathology, and Stroke Unit, Santo Stefano Hospital, Prato, Italy; 12 Stroke Unit, Fazzi Hospital, Lecce, Italy; 13 Acute Cerebrovascular Diseases Unit, Vittorio Emanuele University Hospital, Catania, Italy; 14 Division of Neurology, C. e G. Mazzoni Hospital and Madonna del Soccorso Hospital, Ascoli Piceno, Italy; 15 Neurology - Stroke Care Program, Parma University Hospital, Parma, Italy; 16 University Neurology, Bari Polyclinic Hospital, Bari, Italy; 17 Neurosonology and Stroke Unit, Siena University Hospital, Siena, Italy; 18 Neurovascular Treatment Unit, Tor Vergata Polyclinic Hospital, Rome, Italy; 19 Stroke Unit, C. Mondino Foundation, Pavia, Italy; 20 Neurology, San Iacopo Hospital, Pistoia, Italy; 21 Neurology, Apuane Hospital, Massa e Carrara, Italy; 22 Stroke Unit, Sant’Agostino Estense New Hospital, Modena, Italy; 23 Neurology and Stroke Unit, SS Filippo e Nicola Hospital , Avezzano, Italy; 24 Neurology, San Donato Hospital, Arezzo, Italy; 25 Stroke Center – Neurology, Città Di Castello Hospital and Gubbio-Gualdo Tadino Hospital, Città di Castello, Italy; 26 Neurology, Guzzardi Hospital, Ragusa, Italy; 27 Stroke Unit, Verona University Hospital, Verona, Italy; 28 Neurology, Sant’Andrea Hospital, Rome, Italy; 29 Neurovascular Treatment Unit, Belcolle Hospital, Viterbo, Italy; 30 Neurology, Circolo University Hospital, Varese, Italy; 31 Stroke Unit, Bolzano Hospital, Bolzano, Italy; 32 Neurology, Monserrato University Hospital, Cagliari, Italy; 33 Neurology, Jazzolino Hospital, Vibo Valentia, Italy; 34 Neurology, Da Saliceto Hospital, Piacenza, Italy

**Keywords:** Fabry disease, Genetic disorders, TIA, Ischemic stroke, Intracerebral hemorrhage, Prevention

## Abstract

**Background:**

TIA and stroke, both ischemic and hemorrhagic, may complicate Fabry disease at young-adult age and be the first manifestation that comes to the clinician’s attention. No definite indications have yet been elaborated to guide neurologists in Fabry disease diagnostics. In current practice, it is usually sought in case of cryptogenic strokes (while Fabry-related strokes can also occur by classical pathogenic mechanisms) or through screening programs in young cerebrovascular populations. Data on recurrence and secondary prevention of Fabry’s stroke are scanty.

**Methods:**

The study had a prospective observational design involving 33 Italian neurological Stroke Units. Considering the incidence of TIA/stroke in the European population aged < 60 years and the frequency of Fabry disease in this category (as foreseen by a pilot study held at the Careggi University-Hospital, Florence), we planned to screen for Fabry disease a total of 1740 < 60-year-old individuals hospitalized for TIA, ischemic, or hemorrhagic stroke. We investigated TIA and stroke pathogenesis through internationally validated scales and we gathered information on possible early signs of Fabry disease among all cerebrovascular patients. Every patient was tested for Fabry disease through dried blood spot analysis. Patients who received Fabry disease diagnosis underwent a 12-month follow-up to monitor stroke recurrence and multi-system progression after the cerebrovascular event.

**Discussion:**

The potential implications of this study are as follows: (i) to add information about the yield of systematic screening for Fabry disease in a prospective large cohort of acute cerebrovascular patients; (ii) to deepen knowledge of clinical, pathophysiological, and prognostic characteristics of Fabry-related stroke.

## Introduction

Fabry disease (FD) (OMIM 301500) is an x-inherited glycosphingolipid storage disorder due to mutations in α-galactosidase A (GLA) gene; the deficient enzyme activity determines a multi-organ disease with progressive manifestations. Transient ischemic attack (TIA) and stroke (both ischemic or hemorrhagic) are reported as serious complications in young-adult patients with FD and may lead to relevant morbidity and reduced life expectancy [1]. In young Fabry’s males, stroke frequency has been estimated to be 12 times higher compared with general population [2].

Due to heterogeneity and non-specificity of its symptoms, FD is often unrecognized and screening programs in high-risk populations are increasingly used to facilitate its identification [3].

The effectiveness of this approach is still a matter of debate: along with some well-defined pathogenic mutations (responsible for classical or later-onset Fabry disease), screening programs seem to discover an unexpected high number of genetic variants of unknown significance (GVUS) and polymorphisms of GLA gene, and these findings have proved to be particularly frequent among cerebrovascular patients [4]. FD prevalence in high-risk populations varies between studies in relation to the interpretation that different Authors give to the identified genetic variants [5]. Another factor that may influence the compute of the prevalence is the geographical area of origin of patients: in a recent review, FD has been found in 0.88% of individuals screened for a cerebrovascular disorder in non-Asian and in 0.62% in Asian countries [6].

Due to its relatively low prevalence among stroke populations, a clinical workup for FD is commonly considered appropriate only in case of cryptogenic events in younger patients [7]. However, data on the frequency and mechanisms of Fabry-related stroke can be skewed limiting the focus on cryptogenic rather than all types of stroke. In addition, no prospective data on neurological and multisystem outcomes are available for patients in whom a de novo diagnosis of Fabry disease is made after a stroke.

A nationwide registry (33 neurological stroke units, spread over the entire Italian territory) was set up to diagnose FD among young-adults with a recent cerebrovascular event and to systematically collect information for these purposes.

## Methods

**Fig. 1:**
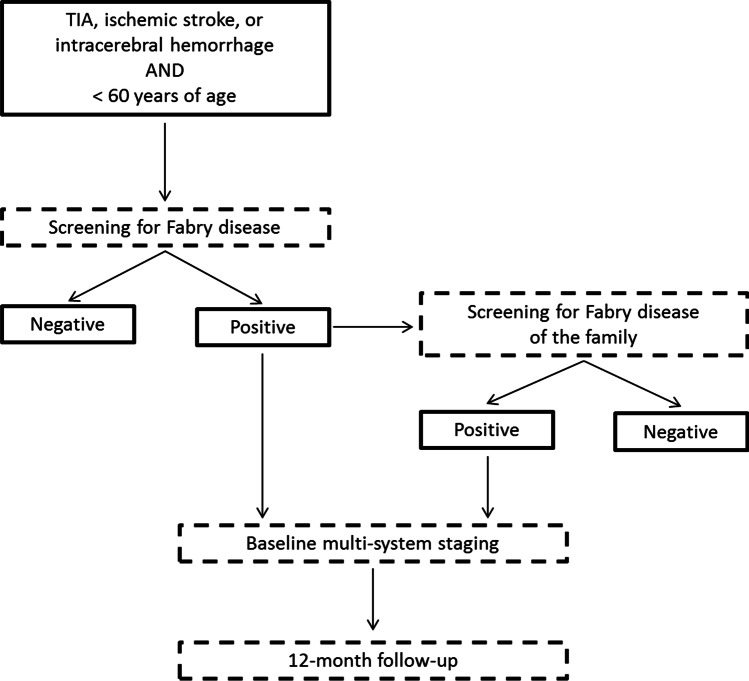
Fabry-Stroke Italian Registry (FSIR) flow-chart. The diagram summarizes the screening and follow-up procedures that cerebrovascular and Fabry patients undergo after the enrollment in the FSIR study

Each patient < 60 years of age admitted for TIA, ischemic stroke (IS), or intracerebral hemorrhage (ICH) was screened for FD and listed in the Cerebrovascular-section of the Fabry-Stroke Italian Registry (FSIR). Patients identified as having a novel FD diagnosis (including individuals with polymorphisms or GVUS) were entered in the Fabry-section of the registry, where a baseline multi-system staging and prospective 12-month follow-up were recorded. Based on a structured history and baseline test results, each FD patient was identified as a previously misdiagnosed case or as a novel diagnosis in which cerebrovascular disorder was the first manifestation of FD. Family members with FD, as identified by the pedigree analysis, were also included in this section through an identification code that links the relative to the index case (specifying the degree of kinship and allowing for a separate analysis of the data) (Fig. 1). We planned to screen for FD a total of 1740 cerebrovascular patients.

## FD screening procedures

A dried blood spot (DBS) test was performed to dose enzyme activity in males and to sequence GLA exonic regions and intron–exon boundaries in females and males with reduced α-galactosidase activity.

## Data collection

Data were entered by local investigators into an electronic case report form accessible at http://www.studiorifs.it with a confidential account. The central database was managed by an experienced data manager (GP) at the NEUROFARBA Department, University of Florence. Before starting enrollment, a detailed manual was distributed to guide collaborators in appropriately applying definitions and accurately registering variables.

Cerebrovascular-section of FSIR included socio-demographics, risk factors, stroke pathogenesis and recurrence, FD-suggestive symptoms, and results of FD testing. TIA/IS etiology was defined using the computerized CCS (Causative Classification of Stroke) algorithm [8]. ICH etiology has been classified according to SMASH-U (Structural lesion, Medication, Amyloid angiopathy, Systemic/other disease, Hypertension, Undetermined) classification [9].

Fabry-section of FSIR included blood and urine laboratory tests, results of cerebral, vascular, and cardiac imaging, and treatments applied for both vascular brain event and FD. Based on a multi-system staging, we computed the Disease Severity Scoring System (DS3) [10]. This is an international standardized scale to grade FD severity after assessing 4 disease domains: peripheral nervous system, kidney, heart, and central nervous system.

The recurrence of cerebrovascular events and the multi-organ progression were recorded at follow-up, revaluating DS3, together with results of blood and urine tests, and cardiac imaging. A 12-month MRI was performed to monitor cerebrovascular progression (including markers of small vessel disease).

## Central imaging assessment

MRI was evaluated centrally at the Careggi Hospital, blind to clinical data. Infarct type, number, and vascular territory were recorded. For ICH, number and deep versus lobar location were evaluated. Markers of small vessel disease were rated using STRIVE criteria [11]. Imaging markers associated with FD were also searched, including pulvinar sign, tortuosity, and/or ectasia of the basilar artery. The basilar artery diameter was measured using the Fellgiebel method [12]. Basilar artery tortuosity was rated according to indications of SIFAP study [13].

## Data monitoring

A monthly monitoring program was performed by the coordinating center, each local investigator being notified about missing or inconsistent data. Automatic alerts about incomplete variables were also provided during the saving procedure of case report forms.

## Sample size estimates

The sample size of 1740 cerebrovascular patients has been calculated considering the following: (i) the incidence of TIA/stroke in the European < 60-year-old population (29/100.000) reported by the European Registers Of Stroke study [14]; (ii) the expected FD prevalence among Italian cerebrovascular patients of the same age (2.8% with 95% CI 0.57–8.18) reported from the pilot FD screening study carried out in Florence in 2011–2012 [15].

Based on these data, we were waiting for 49 (95% CI 10–142) de novo FD diagnoses from the screened population. Assuming that, on average, five family members would be diagnosed as having FD for every proband [16], we could estimate 245 (95% CI 50–710) FD relatives.

## Statistical analyses

In addition to estimating the incidence of Fabry-related stroke in a continuous, prospective, and large cohort of young-adults with acute cerebrovascular disease, the further endpoints were recurrence of cerebrovascular accidents and progression of multi-system involvement on DS3 in FD patients at 12-month follow-up.

According to the literature, the cumulative incidence of stroke recurrence within 1 year is around 3.0% [17]. Considering that these values refer to younger patients (< 45 years) and that stroke frequency is correlated with age, we supposed in our population a higher recurrence rate, reasonably 4%. We also hypothesized that FD patients may have a higher recurrence risk. The population size of our study had a 12% power to identify a frequency of 5% relapses at 1 year in the FD group. The power could increase to 33% if the recurrence rate in FD patients was 7% and to 63% if this was 10% (alpha = 0.05, one-tailed test).

Regarding the progression of multi-system involvement on DS3 at 12 months, the overall forecasted sample size of FD patients offered a power greater than 90% to identify over time changes of at least 2 points at DS3 (alpha = 0.05, two-tailed test).

As secondary endpoint, we investigated the progression of markers of small vessel disease between baseline and 12-month MRI.

## Study organization

The NEUROFARBA Department coordinated and monitored the project. An experienced interpretative committee was established, including experts in the field of FD, lysosomal diseases, stroke management, and advanced statistics, for monitoring both accrual and quality of data and interpreting results of FD screening.

## Discussion

The yield of systematic screening for FD in young patients hospitalized for an acute cerebrovascular disorder remains controversial. One of the advantages of this approach is to facilitate the diagnosis of a rare and phenotypically heterogeneous disease increasing the chance of treating it if revealed. On the other hand, the high number of GLA gene GVUS and polymorphisms detected by screening programs represents a challenging issue for neurologists: according to van der Tol et al., over 90% of individuals tested for stroke or small fiber neuropathy carried an uncertain/neutral or of unknown significance variant of GLA gene [4], whereas Doheny et al. estimated that FD prevalence in populations with ischemic or cryptogenic strokes decreased from 0.67% (males) and 1.11% (females) to 0.13% (males) and 0.14% (females) when only mutations responsible for classical or later-onset phenotype were considered [5].

Whether GVUS are an incidental finding without any clinical significance, a mutation responsible for a non-classical (predominantly neurological) form of FD, or whether they may have some role (independently from FD) in the pathogenesis of the cerebrovascular disease, is a debated challenge.

Since 2008, 4 screening studies for FD have been conducted among patients suffering from a cerebrovascular event in Italy. The frequency of “well-defined” GLA gene mutations was as follows: 3% in the GENS multicenter study, conducted in the Lombardy Region in patients selected by a diagnostic algorithm [18]; 2.8% in the single center study conducted in Florence [15]; 0.6% in the single center study conducted in Northern Sardinia [19]; 0% in the IPSYS multicenter study [20]. In addition, the frequency of polymorphisms and GVUS of the GLA gene was respectively as follows: 0% (GENS) [18], 0.9% (Florence) [15], 0.6% (Northern Sardinia) [19], 0.6% (IPSYS) [20]. These figures look to some extent different from the prevalence rates reported in the most recent literature reviews [5, 6]: although the differences may be the mere expression of a sampling bias, it cannot be excluded that the distribution of this genetic disease may also be influenced by the geographic area of provenience of patients.

Finally, despite the reported negative impact of stroke occurrence on survival and quality of life of FD patients [1], data regarding cerebrovascular involvement in FD remain scanty. In a recent review about FD treatments, only a minority of controlled studies paid attention to the central nervous system (CNS) and information about neurological outcomes derives mostly from observational and retrospective studies, open design of which may be a source of bias regarding clinical events and symptoms type and severity [21].

The CNS outcomes’ sub-investigation in FD was probably due to a shared concept that enzyme replacement therapy (ERT) is not useful because it is unable to cross the blood–brain barrier. However, a recent meta-analysis has documented a beneficial effect of ERT in stroke prevention [22], and the recent availability of a specific chaperone (able to cross the blood–brain barrier) has further contributed to restore interest in this topic.

The multicenter design of FSIR, including an active program for identification of naïve patients with Fabry-related stroke and their follow-up, may reduce inaccuracies and provide more systematic, prospective, and possibly informative data.

## Conclusions

We believe that our study may contribute to generate selective and detailed data about relationships between stroke and FD. From a translational viewpoint, the concept of potential interactions between conventional risk factors and FD angiopathy may have further potential therapeutic implications.

## Data Availability

Access to electronic case report form is available from the corresponding author upon reasonable request.

## References

[1] Mehta A, Clarke JTR, Giugliani R, Elliot P, Linhart A, Beck M, Sunder-Plassmann G, FOS Investigators (2009). Natural course of Fabry disease: changing pattern of causes of death in FOS — Fabry Outcome Survey. J Med Genet.

[2] Sims K, Politei J, Banikazemi M, Lee P (2009). Stroke in Fabry disease frequently occurs before diagnosis and in the absence of other clinical events: natural history data from the Fabry Registry. Stroke.

[3] Linthorst GE, Bouwman MG, Wijburg FA, Aerts JMFG, Poorthuis BJHM, Hollak CEM (2010). Screening for Fabry disease in high-risk populations: a systematic review. J Med Genet.

[4] van der Tol L, Smid BE, Poorthuis BJHM, Biegstraaten M, Lekanne Deprez RH, Linthorst GE, Hollak CEM (2014). A systematic review on screening for Fabry disease: prevalence of individuals with genetic variants of unknown significance. J Med Genet.

[5] Doheny D, Srinivasan R, Pagant S, Chen B, Yasuda M, Desnick RJ (2018). Fabry Disease: prevalence of affected males and heterozygotes with pathogenic GLA mutations identified by screening renal, cardiac and stroke clinics, 1995–2017. J Med Genet.

[6] Lee TH, Yang JT, Lee JD, Chang KC, Peng TI, Changa TY, Huang KL, Liu CH, Ryu SJ, Burlina AP (2019). Genomic screening of Fabry disease in young stroke patients: the Taiwan experience and a review of the literature. Eur J Neurol.

[7] Kolodny E, Fellgiebel A, Hilz MJ, Sims K, Caruso P, Phan TG, Politei J, Manara R, Burlina A (2015). Cerebrovascular involvement in Fabry disease Current Status of Knowledge. Stroke.

[8] Ay H, Benner T, Arsava EM, Smith WS, Sorensen AG, Koroshetz WJ (2007). A computerized algorithm for etiologic classification of ischemic stroke: the Causative Classification of Stroke System. Stroke.

[9] Meretoja A, Strbian D, Putaala J, Curtze S, Haapaniemi E, Mustanoja S, Sairanen T, Satopää J, Silvennoinen H, Niemelä M, Kaste M, Tatlisumak T (2012). SMASH-U: a proposal for etiologic classification of intracerebral hemorrhage. Stroke.

[10] Giannini EH, Mehta AB, Hilz MJ, Beck M, Bichet DG, Brady RO, West M, Germain DP, Wanner C, Waldek S, Clarke JT, Mengel E, Strotmann JM, Warnock DG, Linhart A (2010). A validated disease severity scoring system for Fabry disease. Mol Genet Metab.

[11] Wardlaw JM, Smith EE, Biessels GJ, Cordonnier C, Fazekas F, Frayne R, Lindley RI, O’Brien JT, Barkhof F, Benavente OR, Black SE, Brayne C, Breteler M, Chabriat H, DeCarli C, de Leeuw FE, Doubal F, Duering M, Fox NC, Greenberg S, Hachinski V, Kilimann I, Mok V, van Oostenbrugge R, Pantoni L, Speck O, Stephan BCM, Teipel S, Viswanathan A, Werring D, Chen C, Smith C, van Buchem M, Norrving B, Gorelick PB, Dichgans M, STandards for ReportIng Vascular changes on nEuroimaging (STRIVE v1) (2013). Neuroimaging standards for research into small vessel disease and its contribution to ageing and neurodegeneration. Lancet Neurol.

[12] Fellgiebel A, Keller I, Marin D, Müller MJ, Schermuly I, Yakushev I, Albrecht J, Bellhäuser H, Kinateder M, Beck M, Stoeter P (2009). Diagnostic utility of different MRI and MR angiography measures in Fabry disease. Neurology.

[13] Fazekas F, Enzinger C, Schmidt R, Grittner U, Giese AK, Hennerici MG, Huber R, Jungehulsing GJ, Kaps M, Kessler C, Martus P, Putaala J, Ropele S, Tanislav C, Tatlisumak T, Thijs V, von Sarnowski B, Norrving B, Rolfs A, on behalf of the SIFAP 1 Investigators (2015). Brain magnetic resonance imaging findings fail to suspect fabry disease in young patients with an acute cerebrovascular event. Stroke.

[14] Heuschmann PU, Di Carlo A, Bejot Y, Rastenyte D, Ryglewicz D, Sarti C, Torrent M, Wolfe CDA, European Registers of Stroke (EROS) Investigators (2009). Incidence of Stroke in Europe at the beginning of the 21st century. Stroke.

[15] Romani I, Borsini W, Nencini P, Morrone A, Ferri L, Frusconi S, Donadio VA, Liguori R, Donati MA, Falconi S, Pracucci G, Inzitari D (2015). De novo diagnosis of Fabry Disease among italian adults with acute ischemic stroke or transient ischemic attack. J Stroke Cerebrovasc Dis.

[16] Laney DA, Fernhoff PM (2008). Diagnosis of Fabry disease via analysis of family history. J Genet Couns.

[17] Putaala J, Haapaniemi E, Metso AJ, Metso TM, Artto V, Kaste M, Tatlisumak T (2010). Recurrent ischemic events in young adults after first-ever ischemic stroke. Ann Neurol.

[18] Bersano A, Markus HS, Quaglini S, Arbustini E, Lanfranconi S, Micieli G, Boncoraglio GB, Taroni F, Gellera C, Baratta S, Penco S, Mosca L, Grasso M, Carrera P, Ferrari M, Cereda C, Grieco G, Corti S, Ronchi D, Bassi MT, Obici L, Parati EA, Pezzini P, De Lodovici ML, Verrengia EP, Bono G, Mazucchelli F, Zarcone D, Calloni MV, Perrone P, Bordo BM, Colombo A, Padovani A, Cavallini A, Beretta S, Ferrarese C, Motto C, Agostoni E, Molini G, Sasanelli F, Corato M, Marcheselli S, Sessa M, Comi G, Checcarelli N, Guidotti M, Uccellini D, Capitani E, Tancredi L, Arnaboldi M, Incorvaia B, Tadeo CS, Fusi L, Grampa G, Merlini G, Trobia N, Comi GP, Braga M, Vitali P, Baron P, Grond-Ginsbach C, Candelise L, on behalf of Lombardia GENS Group (2016). Clinical pregenetic screening for stroke monogenic diseases. Results From Lombardia GENS Registry. Stroke.

[19] Fancellu L, Borsini W, Romani I, Pirisi A, Deiana GA, Sechi E, Doneddu PE, Rassu AL, Demurtas R, Scarabotto A, Cassini P, Arbustini E, Sechi G (2015). Exploratory screening for Fabry’s disease in young adults with cerebrovascular disorders in northern Sardinia. BMC Neurol.

[20] Poli L, Zedde M, Zini A, Del Sette M, Lodigiani C, Spalloni A, Di Lisi F, Toriello A, Piras V, Stilo C, Tomelleri G, Tancredi L, Paciaroni M, Silvestrelli G, Adami A, Costa P, Morotti A, Giuli V, Caria F, on behalf of the Italian Project on Stroke in Young Adults (IPSYS) Investigators (2017). Screening for Fabry disease in patients with ischaemic stroke at young age: the Italian Project on Stroke in Young Adults. Eur J Neurol.

[21] Elliott PM, Germain DP, Hilzc MJ, Spada M, Wannere C, Falissard B (2019). Why systematic literature reviews in Fabry disease should include all published evidence. Eur J Med Genet.

[22] Sheng S, Wub L, Nalleballe K, Sharma R, Brown A, Ranabothu S, Kapoor N, Onteddu S (2019). Fabry’s disease and stroke: effectiveness of enzyme replacement therapy (ERT) in stroke prevention, a review with meta-analysis. J Clin Neurosci.

